# LoRaWAN Geo-Tracking Using Map Matching and Compass Sensor Fusion †

**DOI:** 10.3390/s20205815

**Published:** 2020-10-14

**Authors:** Nico Podevijn, Jens Trogh, Michiel Aernouts, Rafael Berkvens, Luc Martens, Maarten Weyn, Wout Joseph, David Plets

**Affiliations:** 1 Department of Information Technology, Ghent University, imec-WAVES, 9052 Ghent, Belgium; nico.podevijn@ugent.be (N.P.); jens.trogh@ugent.be (J.T.); luc1.martens@ugent.be (L.M.); wout.joseph@ugent.be (W.J.); 2 Faculty of Applied Engineering, University of Antwerp, imec-IDLAB, 2000 Antwerp, Belgium; Michiel.Aernouts@uantwerpen.be (M.A.); rafael.berkvens@uantwerpen.be (R.B.); maarten.weyn@uantwerpen.be (M.W.)

**Keywords:** LoRa, localization, positioning, LoRaWAN, TDoA, tracking, map matching, compass, sensor fusion

## Abstract

In contrast to accurate GPS-based localization, approaches to localize within LoRaWAN networks offer the advantages of being low power and low cost. This targets a very different set of use cases and applications on the market where accuracy is not the main considered metric. The localization is performed by the Time Difference of Arrival (TDoA) method and provides discrete position estimates on a map. An accurate “tracking-on-demand” mode for retrieving lost and stolen assets is important. To enable this mode, we propose deploying an e-compass in the mobile LoRa node, which frequently communicates directional information via the payload of the LoRaWAN uplink messages. Fusing this additional information with raw TDoA estimates in a map matching algorithm enables us to estimate the node location with a much increased accuracy. It is shown that this sensor fusion technique outperforms raw TDoA at the cost of only embedding a low-cost e-compass. For driving, cycling, and walking trajectories, we obtained minimal improvements of 65, 76, and 82% on the median errors which were reduced from 206 to 68 m, 197 to 47 m, and 175 to 31 m, respectively. The energy impact of adding an e-compass is limited: energy consumption increases by only 10% compared to traditional LoRa localization, resulting in a solution that is still 14 times more energy-efficient than a GPS-over-LoRa solution.

## 1. Introduction

The Internet of Things (IoT) allows connecting objects to the internet with the use of wireless sensors. Typical use cases are monitoring temperature, humidity, and soil moisture for smart farming applications [1,2], condition monitoring of air cargo [3], or monitoring vital signs of cows in rural areas [4]. Other examples are a bus positioning system [5,6] and asset tracking for logistics [7]. In nearly all cases, the sensor device transmits this information wirelessly to a gateway or access point which has a back-haul to the internet. In this manner, the data can be further processed and visualized. This paper considers the use case of asset tracking.

IoT devices are often powered by batteries, which will sooner or later need replacement, for example, nodes installed in equipment, pallets, or bikes. For economic reasons, a long-range wireless link is also required in order to have a large coverage area with the least amount of access points or gateways. The advent of Low-Power Wide Area Networks (LPWAN) and their deployment in many countries [8] brings benefits at the level of scalability (thousands of sensors per gateway), coverage range (more than 15 km), and power consumption (battery lifetimes up to more than 5 years). The only constraint is the fact that uplink should (in general) be infrequent and limited in the number of payload (information) bytes. Some examples of LPWAN standards are NB-IoT [9], SigFox [10], and LoRaWAN [11].

Many outdoor asset tracking implementations are using a Global Navigation Satellite System (GNSS) receiver to send GPS coordinates over LPWAN. In [5,6], these implementations are used to track city buses. In [3,12], similar GPS-based approaches are used to track air cargo and bikes. Another approach to perform localization is using the network itself to locate sensor nodes without GPS embedded in the node. The basic principle is that when a node send uplink data to the network, the incoming packets’ meta-data such as Time of Arrival (ToA) and Received Signal Strength (RSS) is recorded on the different gateways. The meta-data and gateway locations are then forwarded to a so-called geo-location solver to estimate the location with a suitable algorithm. The output of the solver is then a (Latitude, Longitude) coordinate [13]. The provided location estimates are not as accurate as for GPS (order of a few meters), but an important advantage is that network-based localization (geo-locating) consumes less power on the mobile node compared to integrating a GPS receiver in the node. Another clear advantage of using LPWAN for localization is that only one technology is used for communication and localization, enabling the manufacturing of low cost and compact sensor nodes. Some use cases of geo-locating using LPWAN include tracking of valuable assets during transportation such as railway cars, truck trailers, and containers [7]. The main downside of geo-locating is the relatively low localization accuracy which might not be beneficial for some use cases. Therefore, this paper will focus on improving this performance metric.

**Figure 1:**
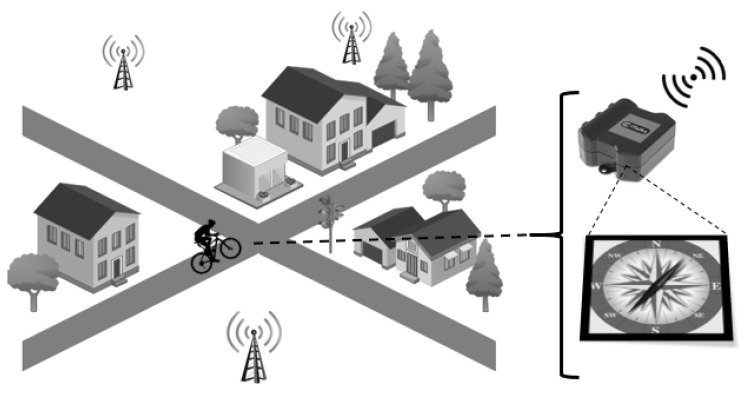
Measurement environment where a LoRaWAN node is attached to a bicycle. The node transmits the e-compass directional data to the network and the Time of Arrival is recorded on all gateways.

In [14], the authors introduced the principle of using a compass on top of LoRaWAN raw location data and tested it for a single route. The goal of this paper is to realize and thoroughly analyze the LoRaWAN Geo-Tracking by the use of map matching and compass sensor fusion. A significant number of test routes is examined for different modes of transportation in different networks, not only in Belgium but also in the Netherlands. Furthermore, the difference between real-time localization and offline a posteriori localization is compared. The main idea is that we take into account the road infrastructure, maximum speed of the node, and (communicated) compass heading. Assuming the tracked item stays on the road network, heading info (e.g., item heading west) can be exploited to exclude other candidate trajectories (e.g., trajectory heading north). The basic setup for this is shown in Figure 1. The novelties of this paper are as follows.

(i) A compass sensor fusion implementation for accurate LoRaWAN localization.
(ii) Improved map matching technique to outperform current available LoRa geo-location possibilities, which works for all LoRa sensor nodes.
(iii) All algorithms are tested with experimental data obtained in different environments and using different modes of transportation (walking, cycling, and driving). The reproducibility of the results is also investigated.

The remainder of the paper is structured as follows. Section 2 presents an overview of related work on LoRa geo-localization methods and improvements proposed in literature. In Section 3, we describe the trajectories and the data collection method, the algorithm implementations, and the different investigated scenarios. Section 4 presents and discusses the results that are obtained using our techniques and compares the energy consumption of our geo-localization solution with GPS-based solutions. Finally, Section 5 summarizes the paper’s findings and provides recommendations for future work.

## 2. Related Work

The most accessible and available way to localize sensor nodes in a LoRaWAN network is by using the RSS metadata that is received by the gateways after an uplink. Such approaches have been studied in our previous works in [15,16]. In [15], the RSS received from up to three gateways was converted to a location using different algorithms, yielding a median accuracy between 1250 m and 2500 m. In case of frequent uplinks and when the mode of transportation is known, map matching further reduced the median error to 700 m. In [16], a mean accuracy around 400 m was obtained with an RSS fingerprinting method based on the collection of a large training database. Similar accuracies (around 350 m) were reported in [17] using other fingerprinting methods.

Current LoRaWAN gateways can accurately determine the Time of Arrival (ToA) of an incoming packet sent by a sensor node. Based on the observed time stamps and the known gateway locations, a Time-Difference-of-Arrival (TDoA) algorithm can estimate the location more accurately than RSS-based methods. Previous research [13,15,18] reported a median accuracy around 200 m when using this method in combination with a maximum likelihood (ML) algorithm. Similar accuracies of around 100 m were reported in [19] for the case of a privately deployed network with static nodes. Improving localization accuracy of static nodes is also investigated in [20], where multiple messages are merged to provide a more accurate estimate. The simulations in [20] showed that it was possible to achieve an average error of less than 100 m using this method. In [21], an improved TDoA algorithm is proposed and compared with a Least Squares (LS) approach. Ninety-five percent percentile values improved from 2200 m to 840 m in a simulated environment. Another approach, correcting the received timestamps of a mobile node by the use of machine learning in combination with stationary reference nodes, reported an accuracy around 61 m [22]. However, it is unclear how many reference nodes are needed, making it potentially unsuitable for large deployments from an economic point-of-view.

This paper will report results from experiments in real public LoRaWAN networks, without using GPS or any other additional infrastructure. Furthermore, it will consider non-stationary nodes and will compare the proposed method with available state-of-the-art TDoA algorithms.

## 3. Materials and Methods

### 3.1. Data Collection and Trajectories

The device for which we estimate its location and trajectory is a LoRaWAN sensor node (MCS iTalks 1608). This device is provisioned on either the Proximus (Belgium) or KPN (Netherlands) LoRa network. The device is configured to have the highest possible uplink frequency possible: every 5 s a 5-byte packet is transmitted on spreading factor (SF) 7. The airtime for this packet is 50 ms: this transmission configuration complies with the ETSI regulations of a 1 percent duty cycle [23]. The transmitted packets are received by the numerous gateways deployed in the outdoor area around the node, after which they are forwarded to the network server. The following data are available:

- A payload (5 bytes) from the device, containing the 5 compass values (also called heading or bearing) recorded during the last 5 s. Due to the unavailability of such a node, the compass values are emulated on a (Samsung Galaxy A20E) smartphone application (OSMTracker for Android), which was carried with the device.
- RSS value received at each gateway with its ID.
- Signal-to-Noise Ratio (SNR) of the received packet at each gateway with its ID.
- Timestamp of the received packet (nanosecond resolution) at each gateway denoted with its ID.

Furthermore, the network topology of all gateways is known for both Proximus and KPN networks (latitude/longitude coordinates for each gateway ID). The recorded data allow processing the data and converting them into the most likely locations and/or trajectory using a suitable algorithm. In order to estimate the localization accuracy, the ground truth (and time) was logged with a GPS application on the smartphone (OSMTracker for Android).

**Figure 2:**
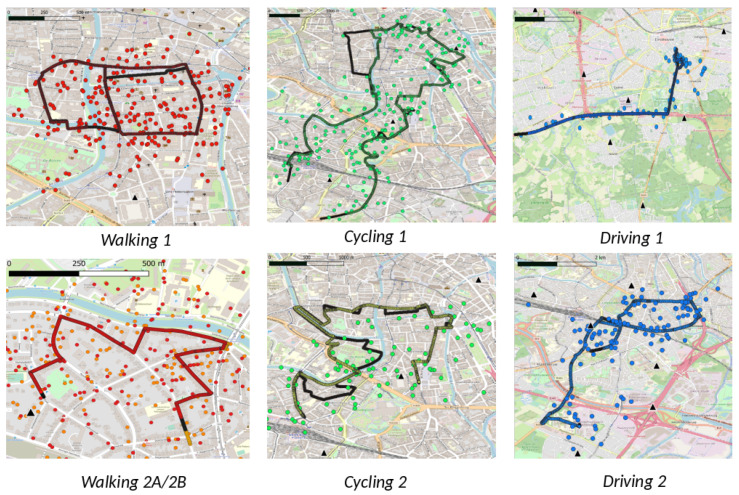
Trajectories with different mobility: left (walking), middle (cycling), and right (driving). The black trace denotes the ground truth. Localization by raw TDoA is shown as coloured dots. Gateways are shown as black triangles. The colored trace is the final result of combining TDoA and absolute compass heading in the map matching algorithm (Scenario OC-Abs. Heading).

The LoRa device and the smartphone were carried in the front pocket of a jacket along 7 trajectories and using different transportation modes. The ground truth trajectories are shown in Figure 2 as a black trace. Each transportation mode (walking, cycling, and driving) was tested twice in a different area. For the second walking trajectory, we traveled the exact same trajectory (noted by “Walking 2A” and “Walking 2B”). Repeating the measurements in the same and/or different areas allows us to check the reproducibility of our results. The characteristics of the trajectories such as duration, velocity and distance are shown in Table 1. Nearly all measurements were performed in Ghent (Belgium) and made use of the Proximus network. The measurements for driving trajectory 1 is the only exception which was done in Eindhoven (Netherlands) and made use of the KPN network.

### 3.2. Algorithm

The algorithms described hereafter were implemented in Python. Algorithm 1 shows the pseudocode of the map matching method with compass sensor fusion. The variables and different steps are discussed in the text below. The algorithm is initialized based on the first TDoA measurement ($TM_{0}$) for which the TDoA solver can calculate a location ($L_{0}$), i.e., if three gateways receive a packet. Next, a predefined number of other locations (MP) are selected around this location and their probability is initialized to one, e.g., the 1000 closest grid points to the current position. This ensures that the algorithm can recover from initially noisy data, e.g., 1000 grid points on a grid with a size of 10 m results in covered surfaces of around 50 hectares (the exact area depends on the density of the road network). This initialization phase forms the starting point of all possible paths that are kept in the memory of the location tracking algorithm (paths).
**Algorithm 1:** Map matching with compass data.
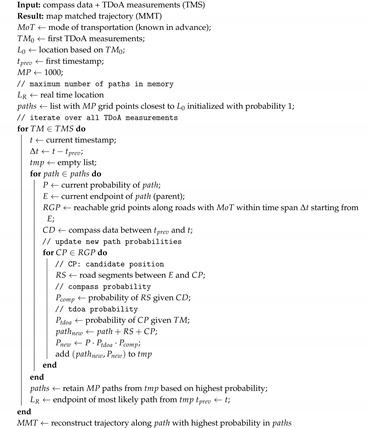


Then, for the subsequent TDoA measurements (TM), all reachable grid positions (RGP) starting from the path’s current endpoint (*E*) are determined for all *paths* in memory, by using the surrounding road network; the elapsed time since last location update (Δt); the known mode of transportation (MoT); and OpenStreetMap metadata, i.e., maximum speed, road type, and one-way information. These reachable grid positions are the candidate positions for the next location update. Transitions between grid points are constrained by the road infrastructure. Each candidate position (CP) retains a link to its parent (i.e., the previous endpoint *E*), a list with visited road segments RS, and a probability that represents this new branch along the road network. This new path (branch) and updated probability are added to the temporary list (tmp) as a tuple $\left(path_{new},\;P_{new}\right)$. The updated probability is the product of the previous probability *P* with a TDoA and compass contribution ($P_{tdoa}$ and $P_{comp}$).

$P_{tdoa}$ is based on the probability of the TDoA measurements given CP, the gateway locations, and the standard deviation of LoRa TDoA measurements, e.g., 383 m in our experimental validation. $P_{comp}$ is based on the probability of the compass data between $t_{prev}$ and *t* given the bearing of the visited road segments RS and is calculated with a standard deviation of 60${}^{{}^{\circ}}$.

The MP paths with the highest probability are retained and serve as input for the next iteration. After all TDoA measurements are processed, the entire trajectory of the path with highest probability in memory is reconstructed. The trajectory is only estimated after all measurements are processed but it is also possible to provide real time location output by retaining the endpoint of the most likely path from tmp in each iteration.

### 3.3. Scenarios

Different scenarios are evaluated, all of which are applicable for different use cases. First, we distinguish between Real-Time (R) Tracking and Offline Tracing (O). In the case of Real-Time Tracking, the localization result is made available immediately after the packet was received. For the case of offline tracing, it is assumed one only requires the fully estimated trajectory at a later instance (this can provide a more accurate trajectory estimation thanks to more data being used). Second, we also differentiate between a device which does not contain a compass (A, for agnostic) and a device that does contain a compass sensor (C). To emulate a device without compass, we ignore the recorded compass data in our algorithms. We denote the agnostic or compass-enabled device by the letters “A” and “C”, respectively. It is therefore possible to combine these considerations into 4 scenarios:

- RA: Real-Time Agnostic: Tracking is needed in real-time and no compass is available in the device
- RC: Real-Time Compass-enabled: Tracking is needed in real-time and compass data are available.
- OA: Offline Agnostic: Offline Tracing at a later instance is needed, the device has no compass data available.
- OC: Offline Compass: Offline Tracing at a later instance is needed with a compass-enabled device.

For each of the 4 scenarios we consider at least 2 methods/algorithms to perform the respective localization and evaluate them for the different trajectories. For the cases that use compass data, there are 2 possibilities. In the first implementation, it is assumed that the compass produces absolute headings. For example, 0 degrees means the asset moves towards the North. This implementation can be used for cases for which it is known how the compass was installed relative to the tracked device, e.g., for tracking bicycles. The second implementation does not rely on this assumption and uses only relative headings, e.g, a transition from a 90 to a 180 degree bearing or from a 30 to a 120 degree bearing means a turn (of 90 degrees) to the right. For example, this method can be used for tracking parcels in transit, where we do not know the absolute orientation of the installed compass node relative to the direction of movement of the tracker. Table 2 gives an overview of all the methods used in this paper.

**Figure 3:**
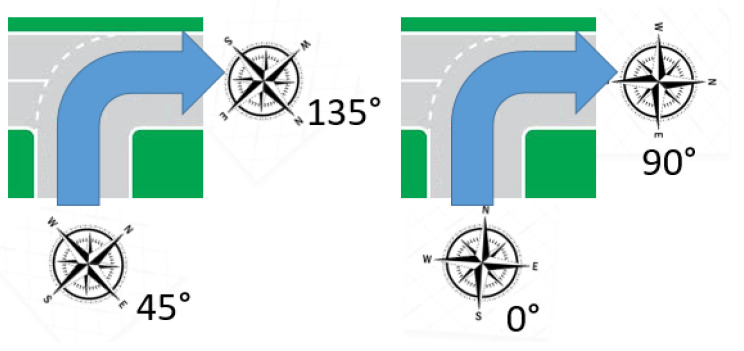
Left figure: compass is misaligned (parcel tracking use case) and heading goes from 45${}^{{}^{\circ}}$ to 135${}^{{}^{\circ}}$. We only know that the relative heading is therefore +90${}^{{}^{\circ}}$, e.g., a turn to the right. Right figure: compass is aligned (bicycle tracking use case) and heading goes from 0${}^{{}^{\circ}}$ to 90${}^{{}^{\circ}}$. We now know the tracker went from South–North to East–West on the road map.

An illustrative example showing the difference between the use of relative and absolute compass headings is shown is Figure 3. The performance of all our implementation methods for the various scenarios is compared to a state-of-the-art commercial solver from Semtech [24], which takes the raw timestamps and gateway locations as inputs.

## 4. Results

### 4.1. Localization Accuracy

**Figure 4:**
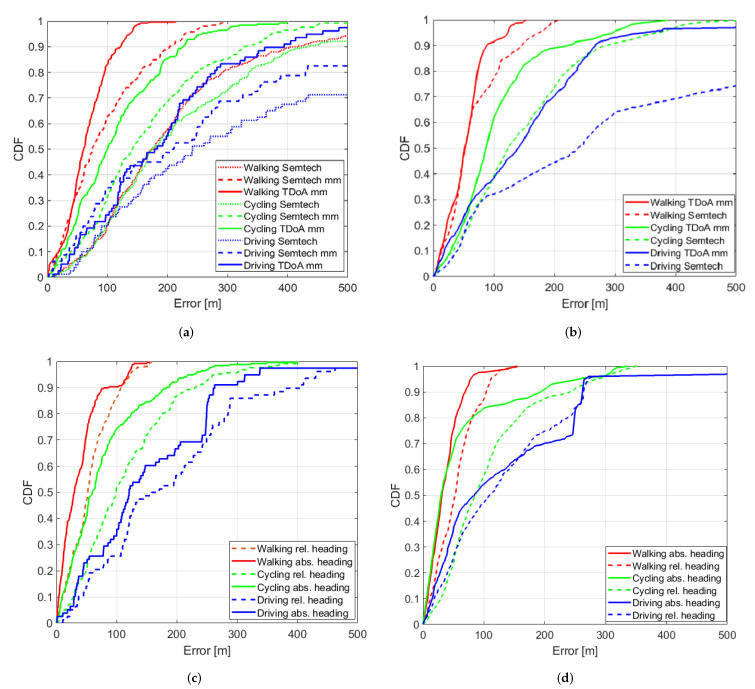
CDFs of the different scenarios evaluated for the walking 1, cycling 1, and driving 1 trajectories. (**a**) Real-time Agnostic (**RA**) scenario: tracking in real-time and no compass is available in the device. (**b**) Offline Agnostic (**OA**) scenario: offline tracing at a later instance, the device has no compass. (**c**) Real-time Compass (**RC**) scenario: tracking is needed in real-time and a compass is available. (**d**) Offline Compass (**OC**) scenario: offline tracing with a compass enabled device.

Figure 4a–d shows the accuracy CDF of the various scenarios, methods, and trajectories. The median (p50) and 90th percentile (p90) accuracy metrics are obtained from each CDF and are summarized in Table 3. The tracking results from method RA/Semtech for the walking, cycling, and driving mobility cases are displayed as dots on the different respective maps in Figure 2. The estimated trajectory of the OC/abs heading scenario is also shown as a colored trace on the maps. Throughout this paper each estimated result (localization on the map, accuracy CDF) is denoted by a different color according to the mobility scenario being evaluated: red corresponds with walking, while green and blue are assigned to cycling and driving. From the maps we can clearly see the improvement made: while the dots give a very rough estimate about the location in real-time, our tracing result nearly overlaps with the ground truth.

Relative improvements (in percentages) when comparing a method to the RA/Semtech traditional localization solver (no compass, no map matching) are shown in Table 4. This table shows that all our methods result in improvements ranging from 1 to 92 percent when compared to the Semtech localization solver. Large improvements are possible, especially when the mobility is low. According to all four CDFs the walking (red lines) did better then the cycling (green lines), which in turn did better than the driving (blue lines) mobility case. This is due to the fact fewer candidate positions are selected for slower modes of transportation (MoT). Regardless of the method used, a minimum improvement of 45 percent is possible for the walking/slow moving case. Therefore, our first recommendation is to let the user supply a mobility motion indicator to each asset to be tracked in case the asset is very likely to be constrained on the road infrastructure (e.g., a bicycle).

When considering the cases for a device without compass (scenarios RA and OA), we see from Table 4 that the best approach is to work with the raw timestamps instead of processed locations for the map matching algorithm: TDoA mm performance is always better than Semtech mm. This is our second recommendation and its impact is also visible in Figure 4a,b.

Table 3 shows that offline tracing (scenarios OA and OC) gives better localization results than their tracking counterparts (scenarios RA and RC). This is due to the fact that when the route is reconstructed all historic data from the start till the end of the measurement are used. Our third recommendation is therefore to implement a feature for which the tracing history can be viewed along with its current most likely localization result.

Relative comparisons for the cases with a compass versus no compass are further shown in Table 5. This table shows that using sensor fusion with a compass improves the localization results: Depending on the mobility a real time median accuracy improvement of up to 70% was possible when the sensor was aligned (which provides an absolute heading = 0 degrees for a moving asset heading South–North). For the reconstructed tracing result, this improvement was up to 72% in the aligned case. When the sensor was not aligned (relative heading) the improvement was up to 35% in the real-time tracking case to 44% for the reconstructed tracing case. The final recommendation is therefore to embed a compass in the LoRa sensor node and align it with the movement direction (if possible).

### 4.2. Discussion

Table 4 shows that regardless of the method used, improvements (between 1% and 92%) are obtained for all seven trajectories. This clearly demonstrates that our methods increase accuracy of the raw output of the Semtech localization solver (“Semtech”), which here serves as reference method. Map matching these raw outputs (“Semtech mm”) always produces better results, with improvements between 14% and 57%. Working with the raw timestamps (TDoA mm) instead of already processed locations in our map matching algorithm gives additional improvements. In Table 4, we note all percentages of TDoA mm are better than Semtech mm regardless if the scenario is offline tracing (OC) or real-time tracking (RA). When adding relative compass data to our TDoA mm algorithm, further improvement of accuracy is shown in Table 5 for nearly all (6/7) trajectories with only 1 exception for the second cycling trajectory (the degradation was only 6 %). When the compass is indicating 0 degrees for a movement from South to North and does not “rotate”, while the tracker (and asset) are in transit (absolute compass) additional improvement is always guaranteed (Table 5) for all trajectories. Table 3 shows that our algorithm provides a median accuracy lower then 85 m while the Semtech solver median accuracy is always higher than 175 m. The same reasoning can be done for 90th percentile error which is always larger than 400 m, while our algorithm guarantees less then 290 m for this metric on all trajectories. The observations are summarized in Table 6.

When repeating the measurement along the exact same trajectory on a different day (“Walking 2A” and “Walking 2B”), we observe two different raw Semtech localization results (orange vs. red dots in Figure 2). After application of our OC/Abs algorithm, the same trajectory is estimated which in turn overlaps the ground truth trace. This clearly demonstrates the reproducibility of the obtained results.

The above analysis shows that the map matching algorithm delivers a first accuracy improvement over the raw data, as it is able to restrict to paths that are physically possible, given the road network topology and speed limits (with margin). Adding compass data further reduces localization errors. Absolute heading data gives information on the estimated travel direction, possibly excluding other candidate paths that remained after map matching. If absolute heading data is not available, relative heading data is still able to detect direction changes. When mapping these direction changes (e.g., a turn right of 60 degrees) to the road network, it is clear that certain trajectories will be more likely than others. Results confirm that the algorithm intelligently makes use of recorded location and heading data, by investigating to what extent these input data match with the different possible trajectories along the road network. It can be expected that results will be better for sparse road networks, as fewer alternatives are available. On the other hand, for grid street plans like in New York, the algorithm might have more trouble distinguishing between two parallel streets, as such grid plan contains only two street directions and allows only 90 degree turns.

### 4.3. Energy Consumption

In this section we briefly compare two common tracking implementations in terms of energy consumption. First, we calculate the energy consumption when a node sends its GPS coordinates using a GNSS receiver over LoRaWAN (GPS-over-LoRa). In the second implementation, we consider the proposed LoRaWAN tracking with compass. For both implementations, we consider a tracking update interval of 5 s and we quantify the energy consumption in mAh. For a node equipped with a GNSS module, the minimum on-time for a location fix is about 1 s [25]. During this acquisition period the receiver typically consumes 30 mA [25]. Therefore, the consumed acquisition energy per location update is

(1) $$E_{GPS}=\frac{1}{3600}*30=0.0083\;\mathrm{mAh}$$

Transmission of 5 bytes on SF7 over LoRaWAN takes 0.05 s airtime while consuming 40 mA of current in a typical transceiver [26]. Therefore, the consumed energy per location update is

(2) $$E_{LoRa}=\frac{0.05}{3600}*40=0.00056\;\mathrm{mAh}$$

The energy consumption for continuous sampling with a (low-power) typical magnetometer sensor [27] is very low and and can be estimated as

(3) $$E_{magneto}=\frac{5}{3600}*0.040=0.000056\;\mathrm{mAh}$$

The energy reduction factor R for our proposed LoRa+compass solution versus a GPS over LoRa implementation is approximately given by

(4) $$R=\frac{E_{GPS-over-LoRa}}{E_{LoRa+compass}}=\frac{E_{GPS}+E_{LoRa}}{E_{LoRa}+E_{magneto}}=14$$

Our approach is thus 14 times more energy efficient than a GPS-over-LoRa solution. Furthermore the price of integrating a magnetometer (e.g., LIS3MDL) is lower when compared to integrating a GNSS receiver (e.g., CAM-M8Q): 0.8€ compared to 13€ (for an order quantity of 500 samples). Although a GPS-based solution will still deliver superior accuracies, the lower cost and energy consumption will make our solution preferable for use cases where positioning accuracy requirements are not so strict (e.g., tracking of truck trailers). Although we only compared two tracking implementations, there are some other solutions such as Wi-Fi scanning and transmitting MAC addresses and signal strength levels of access points in the area over LPWAN, followed by a calculation in a dedicated server (e.g., Skyhook). The accuracy and energy consumption are in between the two earlier investigated approaches. A disadvantage of this method is maintaining an accurate and up to date database with respect to the locations of the access points. For more information on this implementation we refer to the work in [28].

## 5. Conclusions

In this paper, we proposed methods to track and trace assets building on a combination of LoRaWAN technology and a compass sensor. When comparing with a standard TDoA solver (Semtech), we obtained an improvement between 14% and 79% depending on the mobility scenario (walking, cycling, and driving) and whether the result should be immediately available (Real-Time-Tracking) or known at a later instance (Offline-Tracing). These improvements were possible thanks to the map matching algorithm proposed in this paper. Combined with a compass in the sensor node, the bearing information is transmitted periodically to give prevalence to specific paths for which the bearing matches the heading of the road segment. Using this sensor fusion approach, an additional improvement between 1% and 72% was obtained compared to TDoA map matching. The improvement depends on the mobility of the node/asset, usage of the compass node (fixed or rotatable) and whether the result should again be immediately available (Real-Time-Tracking) or known at a later instance (Offline-Tracing). Our best result was obtained for a walking scenario with a fixed (0 degrees means towards “North”) compass for which we obtained a median accuracy of 17 m (an improvement of 91% versus Semtech). All results were obtained from experiments and the reproducibility was verified. Although our results are not as accurate as GPS, we clearly demonstrated that our implemented geo-tracking is 15 times more energy efficient and far less expensive to implement. Further research directions are using the same techniques for LoRaWAN networks which only have RSS values available and future networks which have also Angle of Arrival (AOA) capabilities. Other future work is to expand our implementations to other LPWAN technologies such as SigFox and Narrow-band IoT.

## Figures and Tables

**Table 1:** Characteristics for the 7 mobile trajectories.

Trajectory	Duration	Distance	Avg. Velocity
**Walking 1**	40 min	3.1 km	4.6 km/h
**Walking 2A**	20 min	1.8 km	5.2 km/h
**Walking 2B**	19 min	1.8 km	5.4 km/h
**Cycling 1**	34 min	10.6 km	19 km/h
**Cycling 2**	21 min	6.5 km	19 km/h
**Driving 1**	18 min	16.2 km	55 km/h
**Driving 2**	19 min	8.5 km	26 km/h

**Table 2:** Overview of the different methods used to obtain the real-time estimated locations and/or reconstructed trajectory.

Scenario	Method	Output	Map Matched	Compass Used	Description
**RA**	**Semtech**	Real-Time location	no	no	TDoA solver from Semtech [24]
	**Semtech mm**	Real-Time location	yes	no	solver, next ’snap to road’ [13]
	**TDoA mm**	Real-Time location	yes	no	algorithm 1 with Pcomp=1 and output $L_{R}$
**OA**	**Semtech mm**	Reconstructed Trajectory	yes	no	solver, next estimate trajectory [13]
	**TDoA mm**	Reconstructed Trajectory	yes	no	algorithm 1 with Pcomp=1 and output MMT
**RC**	**Rel. Heading**	Real-Time location	yes	yes-Relative	algorithm 1 with output $L_{R}$
	**Abs. Heading**	Real-Time location	yes	yes-Absolute	algorithm 1 with output $L_{R}$
**OC**	**Rel. Heading**	Reconstructed Trajectory	yes	yes-Relative	algorithm 1 with output MMT
	**Abs. Heading**	Reconstructed Trajectory	yes	yes-Absolute	algorithm 1 with output MMT

**Table 3:** Summary of the obtained $p_{50}$/$p_{90}$ accuracies (in meters) for the various scenarios, methods, and trajectories.

Scenario	Method	Transportation Mode						
		WALK 1	WALK 2A	WALK 2B	CYCLE 1	CYCLE 2	DRIVE 1	DRIVE 2
**RA**	**Semtech**	175/413	187/494	226/501	175/433	197/704	242/1165	206/599
	**Semtech mm**	75/203	102/169	101/209	142/328	159/469	208/574	177/436
	**TDoA mm**	60/122	69/147	58/120	100/226	141/290	181/384	157/421
**OA**	**TDoA_mm**	52/89	39/63	68/120	87/222	147/417	140/268	126/291
	**Semtech_mm**	52/146	48/89	30/175	124/288	169/569	239/881	160/460
**RC**	**Rel. Heading**	52/107	45/149	49/149	99/230	149/319	164/397	154/421
	**Abs. Heading**	30/84	21/137	20/75	60/184	94/280	121/262	148/367
**OC**	**Rel. Heading**	52/104	22/42	41/137	86/241	156/412	109/264	118/359
	**Abs. Heading**	31/69	17/35	19/47	29/193	47/289	84/262	68/227

**Table 4:** Percentual reduction in median positioning error for the various scenarios, methods, and trajectories.

Scenario	Method	Transportation Mode							
		WALK 1	WALK 2A	WALK 2B	CYCLE 1	CYCLE 2	DRIVE 1	DRIVE 2	AVERAGE
**RA**	**Semtech**	Reference	Reference	Reference	Reference	Reference	Reference	Reference	**Reference**
	**Semtech mm**	57	45	55	19	19	14	14	**32**
	**TDoA mm**	66	63	74	43	28	25	24	**46**
**OA**	**TDoA_mm**	70	79	70	50	25	42	39	**54**
	**Semtech_mm**	70	74	87	29	14	1	22	**42**
**RC**	**Rel. Heading**	70	76	78	43	24	32	25	**50**
	**Abs. Heading**	83	89	91	66	52	50	28	**66**
**OC**	**Rel. Heading**	70	88	82	51	21	55	43	**59**
	**Abs. Heading**	82	91	92	83	76	65	67	**79**

**Table 5:** Relative improvement percentage when comparing the scenarios with compass to the absence of such compass.

Scenario	Method	Transportation Mode						
		WALK 1	WALK 2A	WALK 2B	CYCLE 1	CYCLE 2	DRIVE 1	DRIVE 2
**RC vs RA**	**Rel. Heading**	13	35	16	1	−6	9	2
	**Abs. Heading**	50	70	66	40	33	33	6
**OC vs OA**	**Rel. Heading**	0	44	40	1	−6	22	6
	**Abs. Heading**	40	56	72	67	68	40	46

**Table 6:** General observations.

Test Case	True For
All methods better than Semtech	7/7 trajects
Semtech mm better than Semtech	7/7 trajects
TDoA mm better than Semtech mm (RA and OA)	6/7 trajects
Adding relative compass improves results (RC vs RA and OC vs OA)	6/7 trajects
Adding absolute compass improves results (RC vs RA and OC vs OA)	7/7 trajects
p50 Semtech >175 m and p50 OC-Abs <85 m	7/7 trajects
p90 Semtech >400 m and p90 OC-Abs <290 m	7/7 trajects

## Abbreviations

The following abbreviations are used in this manuscript.

GPS	Global Positioning System
LoRaWAN	Long-Range Wide Area Network
TDoA	Time Difference of Arrival
LoRa	Long Range
IoT	Internet of Things
LPWAN	Low-Power Wide Area Network
NB-IoT	Narrowband Internet of Things
GNSS	Global Navigation Satellite System
RSS	Received Signal Strength
ToA	Time of Arrival
ML	Maximum Likelihood
SF	Spreading Factor
SNR	Signal-to-Noise Ratio
CDF	Cumulative Distribution Function
